# Supplementary data analyses for the associations of child maltreatment and diabetes in adulthood and the mediating effect of personality functioning

**DOI:** 10.1016/j.dib.2023.109441

**Published:** 2023-07-22

**Authors:** Sandra Zara, Elmar Brähler, Cedric Sachser, Jörg M Fegert, Winfried Häuser, Lina Krakau, Hanna Kampling, Johannes Kruse

**Affiliations:** a Department of Psychosomatic Medicine and Psychotherapy, Justus Liebig University Giessen, Germany; b Department of Psychosomatic Medicine and Psychotherapy, Johannes Gutenberg University, Mainz, Germany; c Integrated Research and Treatment Center for Adiposity Diseases, Behavioral Medicine Research Unit, University Medical Center Leipzig, Germany; d Department of Child and Adolescent Psychiatry/Psychotherapy, Ulm University, Ulm, Germany; e Department of Psychosomatic Medicine and Psychotherapy, Technische Universität München, Germany; f Department of Internal Medicine I, Klinikum Saarbrücken, Saarbrücken, Germany; g Department for Psychosomatic Medicine and Psychotherapy, Medical Center of the Philipps University Marburg, Marburg, Germany

**Keywords:** Personality functioning, OPD-SQS, Mediation analysis, Child maltreatment, Abuse, Diabetes, Depression/anxiety, Odd ratios (ORs)

## Abstract

In this article, supplementary data analyses regarding the association between different types of child maltreatment (CM) and diabetes as well as mediation analyses examining the role of personality functioning are provided (original research article: ‘Associations of different types of child maltreatment and diabetes in adulthood – the mediating effect of personality functioning: findings from a population-based representative German sample’) (Zara et al., 2023). Analyses are based on a representative sample of the German population (N = 5,041). Data was acquired through a representative survey conducted by the independent research institute USUMA Berlin. CM, personality functioning, a diabetes diagnosis as well as symptoms of depression and anxiety were assessed using self-report questionnaires (CTQ, OPD-SQS, PHQ-4).

Correlation analyses for all used variables were conducted. Independent *t*-tests were performed to examine whether symptoms of depression and anxiety are elevated in patients with diabetes and CM (compared to no CM). Regarding the odd ratios (ORs) calculated to examine the association between types of CM and diabetes as well as mediation analyses investigating the role of personality functioning in these associations, sensitivity analyses with persons ≥ 30 years are provided. The additional analyses are intended to add to the body of research showing that patients with diabetes experience symptoms of depression and anxiety more frequently compared to the general population (Kampling and Kruse, 2020; Chireh et al., 2019; Smith et al., 2018), examine the association between different types of CM and diabetes, and explore the role of personality functioning in the association between CM and diabetes.

**Specifications Table**

**Table:** 

Subject	Psychiatry and Mental Health
Specific subject area	This article investigates diabetes, child maltreatment, symptoms of depression/anxiety and the psychological construct of personality functioning.
Type of data	Table Figure
How the data were acquired	Representative data was acquired via surveys conducted in cooperation with the independent demography research institute USUMA GmbH Berlin in 2016 and 2019, yielding a total of *N* = 5,041 participants. Households within predefined regions were selected by a random route procedure. In households with multiple persons, one person was randomly selected using the Kish grid. After providing informed consent, the selected person was interviewed by a trained employee.
Data format	Raw data Analyzed
Description of data collection	The survey is conceptualized by the University of Leipzig in cooperation with, among others, the University of Giessen and aims at collecting data on different psychological and psychosomatic topics in a sample, which is representative in terms of age, gender and education. Inclusion criteria are sufficient German language skills, an age > 14 and informed consent before taking part in the study.
Data source location	• Institution: Justus-Liebig University Giessen • City/Town/Region: Giessen, Hesse • Country: Germany
Data accessibility	The dataset is made publicly available at Mendeley data. Data identification number: 10.17632/rkw2g38rjp.1 Direct URL to data: https://data.mendeley.com/datasets/rkw2g38rjp/1
Related research article	S. Zara, E. Brähler, C. Sachser, J.M. Fegert, W. Häuser, L. Krakau, H. Kampling, J. Kruse. Associations of different types of child maltreatment and diabetes in adulthood – the mediating effect of personality functioning: findings from a population-based representative German sample. Ann Epidemiol. 78 (2023) 47-53. 10.1016/j.annepidem.2022.12.004

## Value of the Data

• There is little research on the association between child maltreatment (CM) and diabetes. While previous research could demonstrate a general association between CM and diabetes, results were heterogeneous and rarely addressed the necessary separation for different types of abuse and neglect. Hence, we build on those findings and emphasize the importance of investigating CM types in patients with diabetes in detail.
• In addition, we contribute to exploring potentially associated factors for the well-known issue of the very frequent depression and anxiety symptoms in patients with diabetes. We show that both symptoms of depression and anxiety are not only elevated in patients with diabetes but specifically in those who experienced CM compared to those who did not.
• In order to better understand the underlying pathways between CM and diabetes, we included personality functioning as a mediator, and thus, emphasize the role of psychosocial aspects for treating and understanding diabetes.
• These findings add to the evidence of the detrimental and far-reaching effects of CM. Knowledge regarding the impact of CM and its associations with impaired personality functioning can help inform researchers and practitioners in the somatic and psychosocial field.
• Research should extend these findings by considering e.g. frequency and severity of CM or by addressing type 1 or type 2 diabetes separately.

## 1 Objective

This data article provides additional analyses on the association between various types of CM (sexual, physical and emotional abuse and physical and emotional neglect), personality functioning and diabetes in adulthood. Diabetes is one of the most common chronic diseases in the world with a prevalence of around 10% in Germany [1]. It goes along with an increased morbidity and mortality and a decreased quality of life. The highest incidence of diabetes usually occurs in the age group > 75 years, with type 2 diabetes being the most prevalent here, while type 1 diabetes is mainly found in children and adolescents. The original research article provides insights into the elevated risk of diabetes in adulthood when experiencing CM as well as the mediating effect of personality functioning in the association between different types of CM and diabetes. This data article specifies these findings by providing sensitivity analyses for both ORs and mediation analyses in persons ≥ 30 years to account for the unavailable separation of type 1 and type 2 diabetes. Further, this data article reveals higher mental distress (symptoms of depression and anxiety) in people with experiences of CM and diabetes as opposed to people without experiences of CM.

## 2 Data Description

The raw data for all the analyses is publicly available at the Mendeley data repository [2].

Table 1 gives an overview of the correlations between CM, personality functioning, diabetes as well as symptoms of depression and anxiety. All correlations are rather small, but highly significant, except for diabetes and the OPD-SQS subscale ‘self-perception’, where no significant correlation could be observed.

**Table 1:** Correlations between the subtypes of child maltreatment^1^, personality functioning^2^, diabetes^3^ and symptoms of depression and anxiety^4^ including means and standard deviations.

(n = 2,379)	M	SD	sexual abuse	physical abuse	emotional abuse	physical neglect	emotional neglect	personality functioning	self-perception	interpersonal contact	relationship model	PHQ-4
sexual abuse	5.54	(1.97)	-									
physical abuse	5.87	(2.34)	.450*** [.373, .523]	-								
emotional abuse	6.79	(2.97)	.458*** [.397, .518]	.638*** [.591, .683]	-							
physical neglect	7.64	(2.96)	.246*** [.192, .302]	.443*** [.390, .496]	.423*** [.379, .470]	-						
emotional neglect	9.42	(4.38)	.263*** [.211, .313]	.444*** [.402, .487]	.539*** [.499, .579]	.631*** [.603, .659]	-					
personality functioning	10.57	(8.09)	.305*** [.262, .351]	.297*** [.250, .344]	.459*** [.419, .496]	.193*** [.150, .238]	.259*** [.215, .301]	-				
self-perception	1.72	(2.61)	.307*** [.249, .367]	.295*** [.243, .346]	.436*** [.391, .479]	.256*** [.214, .301]	.294*** [.253, .334]	.804*** [.787, .819]				
interpersonal contact	3.35	(3.08)	.261*** [.221, .305]	.264*** [.216, .312]	.398*** [.355, .440]	.189** [.144, .233]	.237*** [.195, .280]	.882*** [.872, .892]	.651*** [.625, .676]			
relationship model	5.49	(3.79)	.229*** [.193, .270]	.218*** [.175, .262]	.357*** [.319, .396]	.083*** [.045, .125]	.158*** [.112, .203]	.866*** [.856, .875]	.499*** [.468, .530]	.623*** [.598, .648]		
PHQ-4	1.37	(2.14)	.260*** [.206, .312]	.278*** [.224, .329]	.379*** [.332, .426]	.240*** [.193, .287]	.253*** [.212, .294]	.556*** [.522, .592]	.608*** [.569, .646]	.492*** [.456, .528]	.369*** [.336, .407]	
diabetes	-	-	.103*** [.033, .167]	.092*** [.044, .146]	.057*** [.013, .105]	.115*** [.021, .073]	.066*** [.024, .107]	.071*** [.035, .112]	.028 [-.011, .071]	.062** [.023, .106]	.081*** [.042, .122]	.080*** [.038, .125]

*Notes.* p-values: * *p* ≤ .05, ** *p* ≤ .01, ***, *p* ≤ .001. point-biserial correlations were computed with 95% bootstrapped confidence intervals based on 1,000 bootstrap samples.

1 Child maltreatment (CM) was measured with the Childhood Trauma Questionnaire (CTQ), for correlation analyses, summated scores for each subscale were computed.

2 Personality functioning was measured with the OPD-Structure Questionnaire (OPD-SQS), for correlation analyses the summated score was used.

3 Diabetes (yes/no) was assessed via self-report.

4 Symptoms of depression and anxiety were measured with the 4 item Patient Health Questionnaire (PHQ-4).

In Table 2, results of the independent *t-*tests are presented, showing that symptoms of depression and anxiety are elevated in patients with diabetes and CM abuse experiences, but not CM neglect experiences.

**Table 2:** Independent *t*-tests for each type of child maltreatment (CM)^1^ to test if symptoms of depression and anxiety^2^ are elevated in patient with diabetes^3^ and experiences of CM compared to patients without CM.

		depression/anxiety				
	N	M	(SD)	*t*-test	*p*-value	Cohen's *d*
sexual abuse						
yes no	33 298	4.36 2.11	(2.60) (2.43)	*t*(329)=5.01	.001^⁎⁎⁎^	0.92
physical abuse						
yes no	37 296	3.65 2.20	(2.58) (2.50)	*t*(331)=3.22	.001^⁎⁎⁎^	0.58
emotional abuse						
yes no	33 300	4.36 2.14	(2.38) (2.47)	*t*(331)=4.93	.001^⁎⁎⁎^	0.90
physical neglect						
yes no	63 111	2.35 1.86	(2.65) (2.30)	*t*(172)=1.26	.208	0.20
emotional neglect						
yes no	27 147	2.81 1.90	(2.40) (2.42)	*t*(172)=1.81	.072	0.38

*Note.* p-values: * *p* ≤ .05, ** *p* ≤ .01, ***, *p* ≤ .001.

1 Child maltreatment (CM) was measured with the Childhood Trauma Questionnaire (CTQ), the presence of CM was assumed when severity was at least *moderate,* dichotomous variables were used for the analyses.

2 Symptoms of depression and anxiety were measured with the Patient Health Questionnaire (PHQ-4).

3 Diabetes (yes/no) was assessed via self-report.

To examine the association of different types of CM and diabetes additional sensitivity analyses with persons ≥ 30 years for odds ratios (ORs) and mediation analyses supporting the original research article were conducted. In Table 3, ORs are displayed. The odds of reporting diabetes in adulthood are elevated for all types of CM, with the highest ORs for physical and emotional abuse (OR = 2.055, OR = 2.049) and the lowest for emotional neglect (OR = 1.225).

**Table 3:** Odd ratios relating various types of child maltreatment^1^ to diabetes^2^ in persons ≥ 30 years.

	diabetes (*n* = 323)		no diabetes (*n* = 3745)			95%-CI	
child maltreatment	n	(%)	n	(%)	Odd ratio	LL	UL
sexual abuse yes (*n* = 244) no (*n* = 3,806)	33 286	(13.5) (7.5)	211 3,520	(86.5) (92.5)	1.925	1.308	2.832
physical abuse yes (*n* = 252) no (*n* = 3799)	36 285	(14.3) (7.5)	216 3,514	(85.7) (92.5)	2.055	1.415	2.984
emotional abuse yes (*n* = 245) no (*n* = 3802)	35 286	(14.3) (7.5)	210 3,516	(85.7) (92.5)	2.049	1.405	2.989
physical neglect yes (*n* = 485) no (*n* = 1502)	63 106	(13.0) (7.1)	422 1,396	(87.0) (92.9)	1.966	1.413	2.736
emotional neglect yes (*n* = 271) no (*n* = 1714)	27 142	(10.0) (8.3)	244 1.572	(90.0) (91.7)	1.225	0.794	1.889

Notes.

1 Child maltreatment (CM) was measured with the Childhood Trauma Questionnaire (CTQ), the presence of CM was assumed when severity was at least *moderate*.

2 Diabetes was assessed via self-report.

**Fig. 1:**
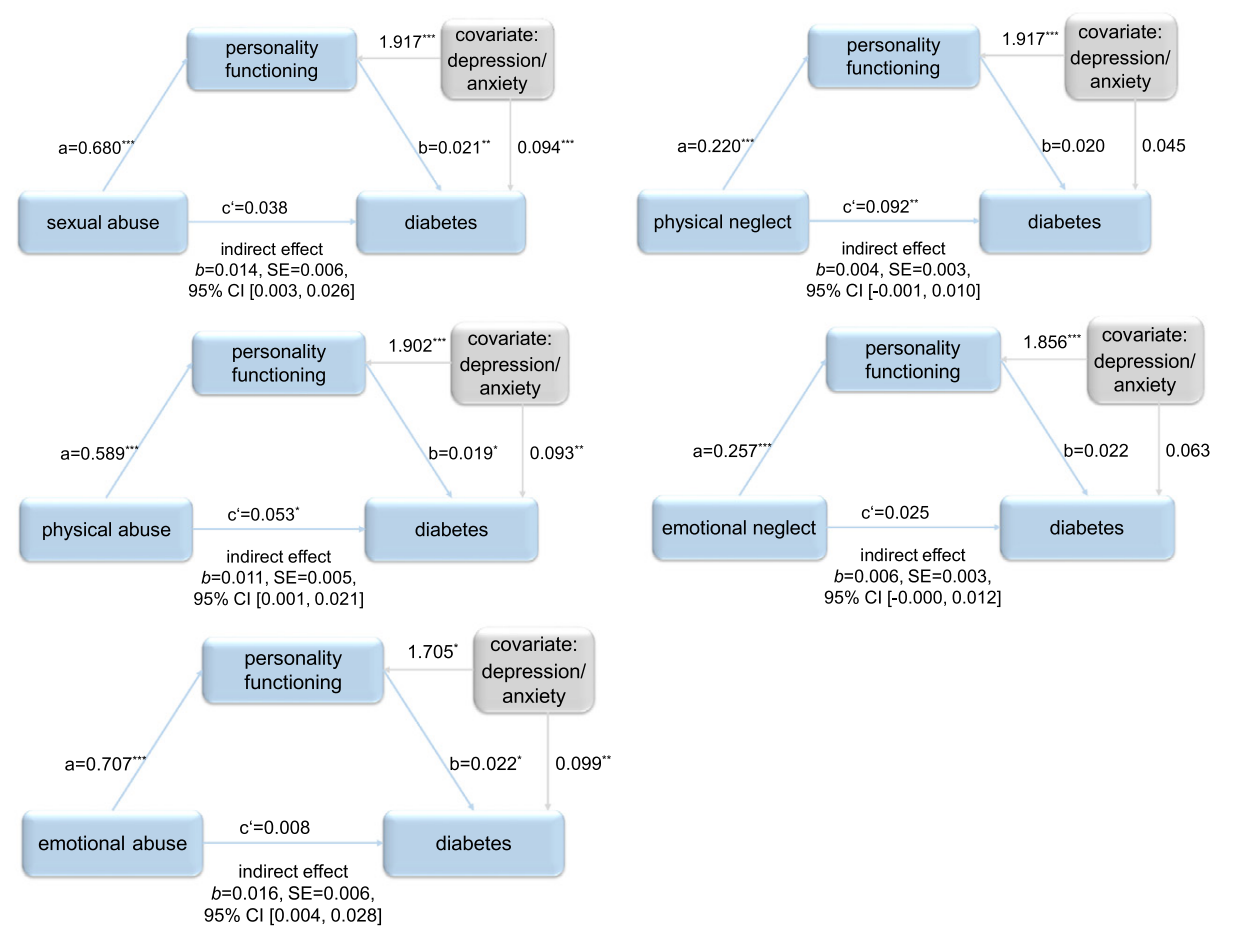
Path diagrams for each mediation analysis conducted, showing the total effect (c’) of the abuse or neglect type of child maltreatment (CM) on diabetes and the indirect (mediating) effect of the abuse or neglect type of CM on diabetes through personality functioning. Conducted with persons ≥ 30 years. p-values: * *p* ≤ .05, ** *p* ≤ .01, ***, *p* ≤ .001.

The results of the mediation analyses conducted with persons ≥ 30 years for each type of CM with personality functioning as mediator and symptoms of depression and anxiety as covariate are shown in Fig. 1. For all CM abuse types, personality functioning mediated the association between CM and diabetes (sexual: *b* = .014, 95% CI [.003, .026]; physical: *b* = .011, 95% CI [.001, .021]; emotional: *b* = .016, 95% CI [.004, .028]). For physical and emotional neglect, no mediating effect of personality functioning could be observed (physical: *b*=.004, 95% CI [-.001, .010], emotional: *b* = .006, 95% CI [-.000, .012]). The proportion mediated (P_M_) of the significant indirect effects amounts to 27.3% for sexual abuse, to 17.4% for physical abuse and to 66.0% for emotional abuse.

## 3 Experimental Design, Materials and Methods

### 3.1 Data Analysis

All statistical analyses were conducted in IBM SPSS Statistics version 28. For the mediation analyses, the PROCESS macro version 4.1 (model 4) by Hayes [3] was used. Correlations were examined using point-biserial correlations suitable for dichotomous and continuous variables, employing 95% bootstrap confidence intervals. To examine if symptoms of depression and anxiety are elevated in patients with diabetes and CM (compared to no CM), the dataset was filtered for all patients with diabetes and without diabetes respectively and *t*-tests employing 95% bootstrap confidence intervals were conducted. Sensitivity analyses regarding ORs and mediation analyses were conducted with persons ≥ 30 to account for the unavailable separation of type 1 and type 2 diabetes. ORs were calculated using dichotomous variables for each CM type and diabetes. For mediation analyses, the summated score of each CM subscale was entered as independent variable, the OPD-SQS summated score was applied as mediator, and diabetes (yes/no) was entered as dependent variable. Symptoms of depression and anxiety (PHQ-4 summated score) were entered as covariate. Bootstrapping was employed to estimate the regression coefficients and the mediation effects were evaluated by examining if the confidence interval of the indirect effect included zero. If not, mediation was evaluated as present. Additionally, for all significant indirect effects, the proportion mediated (P_M_) was calculated [4], [5]. For all analyses, significance level was set at α = .05.

## 4 Materials

### 4.1 Child Maltreatment

Different forms of CM were assessed with the Childhood Trauma Questionnaire (CTQ), which is a widely used screening tool for the assessment of childhood abuse and neglect under the age of 18. Participants answer questions regarding sexual, emotional and physical abuse and emotional and physical neglect on a scale with response options ranging from 1 = “not at all” to 5 = “very often”. Each subscale consists of 5 items, resulting in summated scores for each subscale from 5 (no abuse or neglect in childhood) to 25 (severe abuse or neglect in childhood) and hence, for the total questionnaire from 25 to 125. According to Häuser and colleagues [6] the degree of severity is classified as none to minimal, low to moderate, moderate to severe and severe to extreme. Prevalences are calculated by evaluating CM as present for each person who has at least experienced moderate to severe CM. The German version of the CTQ showed good validity in both the general population and psychiatric patients and good internal consistency, except for physical neglect; sexual abuse: α = .89, physical abuse: α = .80, emotional abuse: α = .87, physical neglect: α = .55 and emotional neglect: α = .83 [7], [8], [9]. In the present study, the CTQ showed good to excellent internal consistency (total: α= 0.87, sexual abuse: α= 0.92, physical abuse: α= 0.84, emotional abuse: α= 0.86, physical neglect: α= 0.55, emotional neglect: α= 0.86).

### 4.2 Personality Functioning

The OPD-Structure-Questionnaire (OPD-SQS) is a self-report questionnaire to screen for participants with deficits in personality functioning. It comprises the three subscales, (1) self-perception, (2) interpersonal contact, and (3) relationship model, with four items on each scale. Response options range from 0 = “does not apply at all” to 4 = “fully applies”, resulting in a summated score ranging from 0 to 48, with higher scores indicating more severe deficits in personality functioning. The OPD-SQS showed good internal consistency (α = .88) [10]. In the present study, internal consistency was acceptable to good (total: α= 0.89, self- perception: α= 0.86, interpersonal contact: α= 0.77, relation-ship model: α= 0.81).

### 4.3 Diabetes

Information on diabetes were obtained through one self-report question in each survey. All participants who answered with ‘yes’ were included as patients with diabetes. Literature suggests high agreement between self-reported diabetes and medical records [11], [12].

### 4.4 Depression and Anxiety Symptoms

To assess symptoms of depression and anxiety, the Patient Health Questionnaire (PHQ-4) consisting of 4 Items was used [13]. It comprises 2 items of the Generalized Anxiety Disorder Scale-2 (GAD-2) to assess anxiety symptoms and the PHQ-2 to assess depression symptoms. Response options range from 0 = “not at all” to 3 = “nearly every day”, resulting in a summated score ranging from 0 to 12, with scores > 6 indicating a probable presence of a depressive or anxiety disorder and scores > 9 indicating a highly probable presence of a depressive or anxiety disorder. The PHQ-4 showed acceptable reliability with McDonald's omega of ω = 0.85 (PHQ-2: ω = 0.77, GAD-2: ω = 0.78) [14]. In the present sample, the internal consistency was good with Cronbach's α = .87.

### 4.5 Methods

**Fig. 2:**
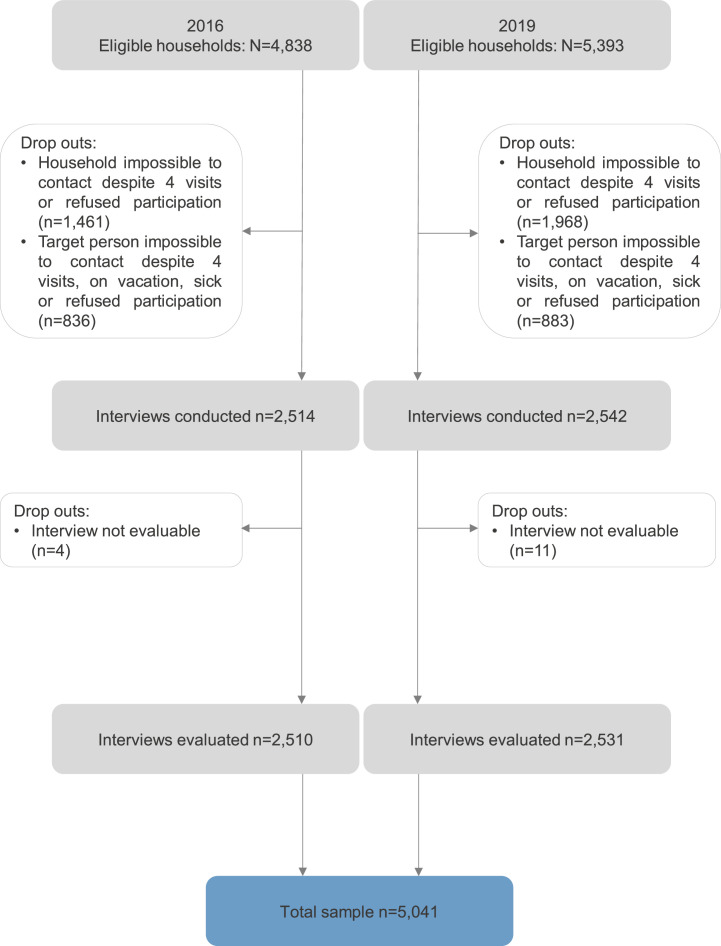
Flow Chart on the number of identified households, included and excluded interviews and the reasons for exclusions.

The data analyzed in this article are based on representative survey data collected in cooperation with the independent demography research institute USUMA GmbH Berlin. The representative surveys of the German population are conducted regularly since 1994, covering a wide range of topics e.g., psychological and physiological burden, societal topics, and quality of life using face-to-face interviews and administering numerous established questionnaires. To collect the data, households within 258 predefined regions are selected by a random route procedure. In households with multiple persons, one person is randomly selected using the Kish-Selection-Grid. Inclusion criteria are sufficient German language skills, an age > 14 and informed consent before taking part in the study. The data contain a weight variable correcting for age, sex and region of living as derived from the German federal statistical office. For the related original article and hence, for the supplementary data analyses presented in this article, data of two independent survey waves (October and November 2016 (N = 2,510) and May and July 2019 (N = 2,531)) containing the relevant measures (sociodemographic data, CTQ, OPD-SQS, diabetes diagnosis, and PHQ-4) were merged, yielding a total of N = 5,041 participants. The response rate of the 2016 survey wave was 51.9%, and for the 2019 survey wave 46.9% (see also Fig. 2). As the investigated effect of impaired personality functioning is believed to play a greater role in patients with type 2 diabetes than type 1 diabetes due to the different pathomechanisms of the diabetes types, we excluded a proportion of participants to minimize the probability of type 1 diabetes and replicated the analyses in an age group over 30 years.

Some methodological limitations of this study have to be considered before interpreting the results. First, the cross-sectional nature of the data does not allow for conclusions regarding cause and effect in the association between different types of CM, personality functioning and diabetes in adulthood. Secondly, additional control variables as indicators for health, such as BMI, smoking or alcohol consumption were not available. Important sociodemographic variables influencing the occurrence of CM were not included in the dataset. Further, all variables were assessed via self-report which might pose a bias. It must be acknowledged that research suggests self-reported diabetes to be fairly reliable and the impact of subjective and objective CM on psychopathology is comparable, likely reducing the risk of false answers [11], [12], [15].

## Ethics Statements

The surveys were conducted in accordance with the Declaration of Helsinki and fulfilled the ethical guidelines of the International Code of Marketing and Social Research Practice of the International Chamber of Commerce and the European Society of Opinion and Marketing Research. Written informed consent was obtained from each participant and the ethical approval was obtained by the Ethics Committee of the Medical Faculty of the University of Leipzig for each study (no. 297/16-ek & no. 145/19‐ek).

## CRediT Author Statement

EB designed the survey, collected the data, and obtained the ethics approval. JK, JMF, WH and CS contributed to data collection. SZ and HK participated in the research design. SZ conducted the data analyses, interpreted the data and wrote the original draft of the manuscript. HK contributed to the writing of the manuscript. HK and JK supervised the drafting of the manuscript. JK, LK, EB, JMF, WH and CS reviewed and edited the manuscript; all authors approved the final version of the manuscript.

## Data Availability

Supplementary data analyses for the associations of child maltreatment and diabetes in adulthood and the mediating effect of personality functioning (Original data) (Mendeley Data).

## Appendix Supplementary materials

Image, application 1
