# Supplementary material for: Supplementary data analyses for the associations of child maltreatment and diabetes in adulthood and the mediating effect of personality functioning
Source: Data Brief. 2023 Jul 22;49:109441. doi: 10.1016/j.dib.2023.109441 (PMC10415691; doi:10.1016/j.dib.2023.109441)
Supplement: Supplementary file 1 [file mmc1.zip › CTQ.pdf]

Childhood Trauma Questionnaire (CTQ)

No	When I was growing up…	never true	rarely true	some times true	often true	very often true
1	I didn’t have enough to eat					
2	I knew that there was someone to take care of me and protect me					
3	People of my family called me things like ‘stupid’, ‘lazy’ or ‘ugly’					
4	My parents were too drunk or high to take care of the family					
5	There was someone in my family who helped me feel that I was important or special					
6	I had to wear dirty clothes					
7	I felt loved					
8	I thought that my parents wished I had never been born					
9	I got hit so hard by someone in my family that I had to see a doctor or go to the hospital					
10	There was nothing I wanted to change about my family					
11	People in my family hit me so hard that it left me with bruises or marks					
12	I was punished with a belt, a board, a cord, or some other hard object					
13	People in my family looked out for each other					
14	People in my family said hurtful or insulting things to me					
15	I believed that I was physically abused					
16	I had the perfect childhood					
17	I got hit or beaten so badly that it was noticed by someone like a teacher, neighbor, or doctor					
18	I felt that someone in my family hated me					
19	People on my family felt close to each other					
20	Someone tried to touch me in sexual way, or tried to make me touch them					
21	Someone threatened to hurt me or tell lies about me unless I did something sexual for them					
22	I had the best family in the world					
23	Someone tried to make me do sexual things or watch sexual things					
24	Someone molested me					
25	I believe that I was emotionally abused					
26	There was someone to take me to the doctor if I needed it					
27	I believed that I was sexually abused					
28	My family was a source of strength and support					

D. P. Bernstein *et al.*, “Development and validation of a brief screening version of the Childhood Trauma Questionnaire,” *Child Abuse & Neglect*, vol. 27, no. 2, pp. 169–190, Feb. 2003, doi:10.1016/S0145-2134(02)00541-0.
