# Supplementary material for: Supplementary data analyses for the associations of child maltreatment and diabetes in adulthood and the mediating effect of personality functioning
Source: Data Brief. 2023 Jul 22;49:109441. doi: 10.1016/j.dib.2023.109441 (PMC10415691; doi:10.1016/j.dib.2023.109441)
Supplement: Supplementary file 1 [file mmc1.zip › OPD-SQS.pdf]

Self-description Questionnaire

OPD-SQS

On the following pages you will find a number of statements that describe various characteristics of a person. Please indicate to what extent these statements apply to you. Please tick the answer which describes you the best in general. There are no right or wrong answers because people differ in the way they perceive themselves. Some statements apply to relationships. Please answer those questions according to how you usually see yourself in relationships. If you have not yet been in a romantic relationship, imagine how you would see yourself in one.

		**fully disagree**	**partly disagree**	**neither agree nor disagree**	**partly agree**	**fully agree**
1.	I sometimes feel like a stranger to myself.	☐	☐	☐	☐	☐
2.	If I think too much about myself, I tend to get confused.	☐	☐	☐	☐	☐
3.	It can be dangerous to let others get too close to you.	☐	☐	☐	☐	☐
4.	I find it difficult to make others understand me.	☐	☐	☐	☐	☐
5.	There is often such a chaos of feelings inside me that I couldn’t even describe it.	☐	☐	☐	☐	☐
6.	I sometimes misjudge how my behaviour affects others.	☐	☐	☐	☐	☐
7.	If others know a lot about me I often feel somehow controlled or observed.	☐	☐	☐	☐	☐
8.	Sometimes my feelings are so intense that I get scared.	☐	☐	☐	☐	☐
9.	I’ve been hurt badly because I misjudged someone.	☐	☐	☐	☐	☐
10.	I find it hard to get in contact with other people.	☐	☐	☐	☐	☐
11.	I don’t have good self-esteem.	☐	☐	☐	☐	☐
12.	My experience is: If you trust people too much you can get nasty surprises.	☐	☐	☐	☐	☐
