# Supplementary material for: Supplementary data analyses for the associations of child maltreatment and diabetes in adulthood and the mediating effect of personality functioning
Source: Data Brief. 2023 Jul 22;49:109441. doi: 10.1016/j.dib.2023.109441 (PMC10415691; doi:10.1016/j.dib.2023.109441)
Supplement: Supplementary file 1 [file mmc1.zip › PHQ-4.pdf]

PHQ-4: THE FOUR-ITEM PATIENT HEALTH QUESTIONNAIRE FOR ANXIETY AND DEPRESSION

Over the last two weeks, how often have you been bothered by the following problems?	Not at all	Several days	More than half the days	Nearly every day
Feeling nervous, anxious or on edge	0	1	2	3
Not being able to stop or control worrying	0	1	2	3
Feeling down, depressed or hopeless	0	1	2	3
Little interest or pleasure in doing things	0	1	2	3
TOTALS				

Total score is determined by adding together the scores of each of the 4 items.
Scores are rated as normal (0-2), mild (3-5), moderate (6-8), and severe (9-12).
Total score ≥3 for first 2 questions suggests anxiety.
Total score ≥3 for last 2 questions suggests depression.

Reprinted with permission from Kroenke K, Spitzer RL, Williams JB, Löwe B. An ultra-brief screening scale for anxiety and depression: the PHQ-4. Psychosomatics. 2009;50(6):613-21. From Principles of Neuropathic Pain Assessment and Management, November 2011.

The PHQ-4 and other tools are available online at www.oregonpainguidance.org/clinical-tools.
